# Six Papers by Lord Lister

**Published:** 1921-03-26

**Authors:** 


### SIX PAPERS BY LORD LISTER.

Not only to all medical libraries will Sir Rickman Godlee's small volume entitled "Six Papers by Lord Lister,"* find a ready welcome, but the short biography and explanatory notes provided form attractive reading to all who take interest in scientific progress and its benefits to mankind. The author's purpose is rather to fix Lister's place in history, than to improve upon the detailed pictures already given of his life.

Great as popular appreciation of Lister has been, he, who always paid the highest tribute to discoveries by others, would have been the first to urge that much of the progress associated with his name, should be pictured as the work of the group rather than the individual. There is, for example, that of Pasteur, five years Lister's senior. Possibly because Pasteur was a chemist, although he extended his investigations far beyond the limits of this science, yet their real applications to medicine were apparently unrecognised by all except Lister. His first attempts to turn Pasteur's discoveries in fermentation towards practical surgery, ultimately aseptic surgery, are described in Paper No. 3 on p. 93, and if the reader tries for one moment to put himself in Lister's place, inspection of this paper is extraordinary fascinating.

It was written at the height of his career, at a time when in charge of some of the most unhealthy and septic wards in the kingdom. In them, compound fractures almost always resulted in the loss of the limb. Fighting this appalling sepsis, Lister's first ideas were that the air teemed with suppurative germs and that the air was therefore the great source of infection. With Pasteur's teaching in his mind he pondered how he should kill the germs already there as well as prevent the entrance of others. If a successful method were devised, he realised that it would revolutionise surgery. Yet he had only crude carbolic acid to work with. His paper shows a series of difficulties and some failures. But let the modern critic bear in mind that little of bacteriology and nothing of the defensive action of living tissues was then known. This work by Lister may have started a technical contest which after the lapse of fifty years is still not truly settled, but it brought to light the principle of anti-bacterial treatment.

Many though the later modifications have been, the reader must see for himself how the antiseptic principle has been the true source of them all. First the strength of antiseptic dressings was reduced, out of respect to the tissues' power of resistance. The reason for the carbolic spray in a supposedly germ-laden atmosphere was obvious. Its abandonment was simply due to gradually accumulating evidence which its employment brought out. The natural defensive powers of the body were proved, and gradually the world realised "aseptic" as opposed to "antiseptic" surgery. In the strictest sense, Lister may have been wrong. In the last stages of the late war, when free excision of damaged tissues, copious ablutions and immediate closure of even large wounds were often followed by primary healing; these methods formed a final demonstration of a greater truth. But for the path by which it was attained we are for ever indebted to the genius of Lister, and the little volume before us should well serve its purpose of making "the modern school" remember.

* By Sir Rickman Godlee, Bart., K.C.V.O., M.S. Published by John Bale, Sons & Danielsson. Price 10s. net.

